# Design, Synthesis and Biological Activity Evaluation of Arylpiperazine Derivatives for the Treatment of Neuropathic Pain

**DOI:** 10.3390/molecules16075785

**Published:** 2011-07-07

**Authors:** Yin Chen, Guan Wang, Xiangqing Xu, Bi-Feng Liu, Jianqi Li, Guisen Zhang

**Affiliations:** 1 Department of Systems Biology, Huazhong University of Science and Technology, 1037 Luoyu Road, Wuhan, 430074, China; 2 Shanghai Institute of Pharmaceutical Industry, 1111 North Zhongshan Road, Shanghai, 200437, China; 3 Jiangsu Nhwa Pharmaceutical Corporation, Ltd. 69# Minzhu South Road Xuzhou City, Jiangsu, 221009, China

**Keywords:** arylpiperazine, antinociceptive, neuropathic pain, spared nerve injury, chronic constriction injury

## Abstract

In this work, a series of arylpiperazine derivatives were synthesized and screened by *in vivo* pharmacological trials. Among the tested compounds, 2-(4-(3-(trifluoromethyl)phenyl)piperazin-1-yl)-1-phenylethanone (**18**) and *2-(4-(2,3-*dimethylphenyl)piperazin-1-yl)-1-phenylethanone (**19**) exhibited potent analgesic activities in both the mice writhing and mice hot plate tests. They showed more than 70% inhibition relative to controls in the writhing test, and increased latency by 116.0% and 134.4%, respectively, in the hot plate test. Furthermore, compound **18** was also active in the models of formalin pain and neuropathic pain without sedative side effects.

## 1. Introduction

Neuropathic pain is a chronic, debilitating pain state that results from injury to the peripheral or central nervous system. It can be triggered by a variety of events or conditions, including diabetes, shingles, and chemotherapy [1,2]. Currently neuropathic pain is usually treated with a variety of drugs, including opioids, non-steroidal anti-inflammatory drugs (NSAIDs) and analgesic adjuvants [3]. As is well-known, opioids induce a wide variety of side effects, including sedation, constipation, respiratory depression, drug tolerance and physical dependence etc [4]. Meanwhile, NSAIDs show adverse reactions at the gastrointestinal level together with inhibition of platelet aggregation and renal toxicity [5]. The so-called “analgesic adjuvants”, e.g., antidepressants, anticonvulsants and anesthetics [5,6,7], show some efficacy in the treatment of neuropathic pain [6,7,8]. However, these analgesic adjuvants have also shown limited effectiveness for neuropathic pain [9]. Therefore, the design and development of novel analgesic agents that can effectively cure neuropathic pains without inducing side effects remains a major challenge in biomedical research [10].

**Figure 1:**
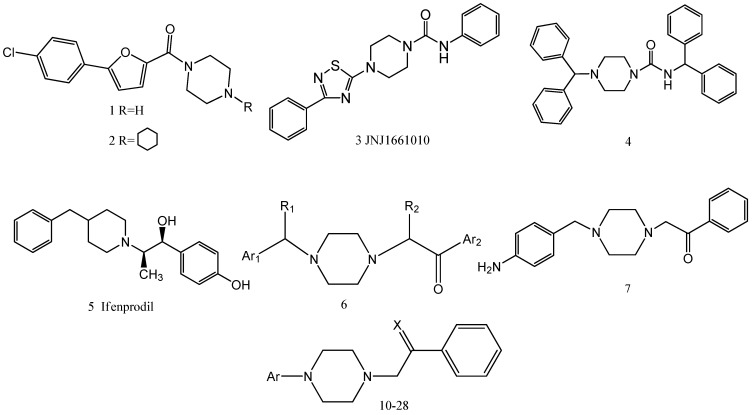
Title and reference compounds.

Piperazines are an important class of chemical compounds with a broad spectrum of biological activities [11,12,13], e.g., anti-infective, anti-cancer, anti-psychiatry, and antinociceptive. Compounds **1** and **2** (Figure 1) demonstrate favorable *in vivo* efficacy in the spinal nerve ligation model of neuropathic pain [14]. Compound **3** (JNJ1661010) attenuates tactile allodynia in the rat mild thermal injury model of acute tissue damage and in the rat spinal nerve ligation model of neuropathic pain [15]. Compound **4** is identified as a potent orally available N-type calcium channel blocker and exhibited mechanical and thermal analgesic activity in the rat spinal nerve ligation model [16]. Thus, piperazine derivatives are obviously attractive candidates for developing novel analgesic drugs.

**Scheme 1:**
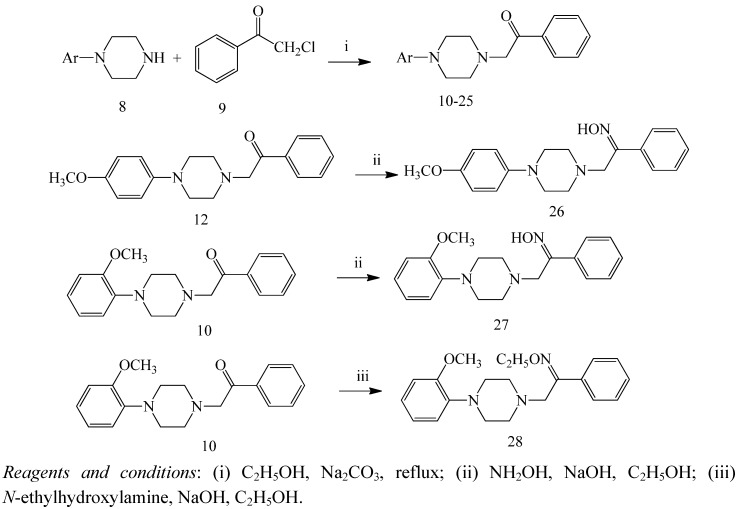
Synthesis of the compounds 10-28.

Ifenprodil (5) was one of the first generation selective NMDA receptor antagonists, which have been used as neuroprotective agents and peripheral expansion of drug for neuropathic pain [17]. In a previous work, a series of aralkylketone piperazine derivatives **6** were synthesized by referring to the active sites of ifenprodil, indicating potent analgesic activities *in vivo* [18]. Subsequent studies showed that SIPI5047 (7) had significant *in vivo* analgesic activities，similar to morphine. However, it induced a major sedative side effect of the nervous system [19]. In this work, we showed that removal of the carbon atom between Ar_1_ and the nitrogen atom of these compounds reduced the sedative side effects without losing analgesic efficacy. To further investigate the structural requirements for drugs capable of improved antinociceptive effects and reduced the sedative side effects, we designed compounds **10-28** (Scheme 1) based on the influence of a number of structural characteristics on antinociceptive effects, including: substituent with different electronic properties on the Ar aromatic ring, replacement of Ar with heterocycle moieties, replacement of carbonyl with the C=N-OR group.

## 2. Results and Discussion

### 2.1. Chemistry

Scheme 1 summarizes the routes for synthesizing compounds **10-28**. Briefly, 2-chloro-1-phenylethanone were reacted with the arylpiperazined in the presence of Na_2_CO_3_ to yield compounds **10-25** [20], compounds **10** and **12** were then transformed into compounds **26-28** by reacting with hydroxylamine hydrochloride or *N*-ethylhydroxylamine hydrochloride. The compounds **10-28** were characterized by their physical and spectral data (^1^H-NMR and mass spectrometry).

### 2.2. Hot Plate Test and Writhing Test

Initially, the analgesic activities of compounds **10-28** were screened by both the acetic acid induced writhing test [21] and the hot plate test [22]. All compounds were administered po, and their efficacies were compared with acetylsalicylic acid and morphine. In the acetic acid induced writhing test, compounds **10**, **11**, **14**, **15**, **18**, **19**, **20**, **24** and **25** were able to reduce the number of writhes while in contrast, other compounds could not reduce the number of writhes (Table 1). Compounds **18** (40 mg/kg) and **19** (10 mg/kg) were found to yield the best analgesic activities with 78.7% and 75.2% inhibition, respectively. It should be noted that compound **18** exhibited more than 70% inhibition at the doses of 10, 20 and 40 mg/kg, which was equivalent to acetylsalicylic acid. To further investigate the antinociceptive profile of compounds **10-28**, a hot plate test was then conducted. Compounds **10**, **18** and **19** increased latency by 191.5%, 116.0% and 134.4%, respectively, while compounds **11**, **14**, **15**, **20**, **24** and **25** showed negative results in the hot plate test (Table 1).

**Table 1:** Antinociceptive effect of final compounds in the writhing test and hot plate test.

Compound ^a^	Ar	R	Dose (mg/kg)	Licking latency (s) ^b^			Writhing	
				Before treatment	After treatment (60 min)	Increased rate of latency (%)	Writhes (per 15 min)	Inhibition (%)
10	2-OCH_3_-Ph	-	10	14.1 ± 1.3	29.0 ± 0.9 *	105.7	12.1 ± 1.1	43.3
			20	13.0 ± 0.9	37.9 ± 1.3 *	191.5	13.2 ± 3.9	38.0
			40	13.7 ± 1.5	29.4 ± 1.5 *	114.6	6.4 ± 2.2 **	70.0
11	3-OCH_3_-Ph	-	10	15.5 ± 0.6	16.7 ± 2.1	7.7	7.2 ± 3.6 *	66.4
			20	17.3 ± 0.9	19.0 ± 1.2	9.8	24.4 ± 9.9	-14.5
			40	18.4 ± 1.3	27.4 ± 4.3	48.9	4.9 ± 1.3 *	77.1
12	4-OCH_3_-Ph	-	10	18.3 ± 1.2	14.0 ± 0.8	-23.5	17.9 ± 4.2	15.8
			20	14.5 ± 1.5	17.0 ± 1.2	17.2	19.7 ± 2.8	7.3
			40	12.4 ± 1.4	22.0 ± 0.9	77.4	20.2 ± 2.9	5.0
13	2-Cl-Ph	-	10	15.6 ± 1.2	18.4 ± 1.6	17.9	11.9 ± 2.7	44.3
			20	19.9 ± 1.3	20.2 ± 2.1	1.5	14.8 ± 3.6	30.5
			40	15.2 ± 2.1	17.6 ± 2.3	15.8	13.0 ± 4.4	38.9
14	3-Cl-Ph	-	10	15.9 ± 1.6	13.9 ± 1.2	-12.6	9.6 ± 3.8	55.0
			20	20.2 ± 2.1	23.1 ± 1.3	14.3	7.2 ± 3.4 *	66.4
			40	18.1 ± 0.9	25.3 ± 2.3	39.8	6.3 ± 2.2 *	70.2
15	4-Cl-Ph	-	10	16.0 ± 1.7	15.8 ± 2.3	-1.3	5.5 ± 1.3 *	74.1
			20	15.5 ± 1.6	22.1 ± 1.3	42.6	8.9 ± 1.0	58.0
			40	18.9 ± 1.4	27.1 ± 3.4	43.4	3.7 ± 1.5 *	82.4
16	2,3-di-Cl-Ph	-	10	17.4 ± 1.3	17.9 ± 1.8	2.9	9.8 ± 3.6	54.1
			20	19.5 ± 1.5	23.3 ± 2.1	19.5	12.2 ± 2.6	42.6
			40	18.3 ± 1.6	26.6 ± 3.9	45.4	28.6 ± 6.6	-34.4
17	4-F-Ph	-	10	15.5 ± 1.8	17.3 ± 0.9	11.6	32.3 ± 3.2	-51.8
			20	17.3 ± 1.9	23.7 ± 1.5	37.0	25.2 ± 5.7	-18.3
			40	18.4 ± 2.1	19.8 ± 2.6	7.6	27.5 ± 2.9	-29.2
18	3-CF_3_-Ph	-	10	17.6 ± 1.3	27.5 ± 2.6	56.3	5.8 ± 2.0 *	73.0
			20	19.2 ± 2.4	39.8 ± 2.4 *	107.3	5.3 ± 1.5 *	75.2
			40	16.2 ± 2.3	35.0 ± 3.1 *	116.0	4.5 ± 1.4 *	78.7
19	2,3-di-CH_3_-Ph	-	10	19.3 ± 1.3	26.6 ± 3.6	37.8	5.3 ± 2.0 *	75.2
			20	15.7 ± 1.4	33.2 ± 1.8 *	111.5	7.7 ± 2.2 *	63.7
			40	16.3 ± 2.3	38.2 ± 2.1 *	134.4	10.3 ± 3.2	51.8
20	6-methoxy-benzo[d]-thiazole	-	10	19.6 ± 2.8	21.0 ± 2.3	7.1	6.9 ± 2.0 *	67.6
			20	16.9 ± 1.6	18.1 ± 2.4	7.1	11.2 ± 3.3	47.2
			40	18.0 ± 1.9	26.4 ± 3.1	46.7	7.3 ± 1.8 *	65.7
21	6-methyl-benzo[d]-thiazole	-	10	18.7 ± 1.6	19.1 ± 3.1	2.1	11.4 ± 2.4	46.3
			20	17.6 ± 2.8	16.3 ± 1.5	-7.4	15.9 ± 4.8	25.9
			40	16.2 ± 2.3	24.1 ± 1.6	48.8	9.3 ± 5.7	56.5
22	4-methyl-benzo[d]-thiazole	-	10	15.0 ± 1.3	21.9 ± 2.1	46.0	32.5 ± 6.1	-52.8
			20	15.9 ± 3.1	17.1 ± 2.6	7.5	12.6 ± 3.7	40.7
			40	18.5 ± 1.8	22.5 ± 2.4	21.6	9.5 ± 4.2	55.6
23	6-chloro-benzo[d]-thiazole	-	10	16.3 ± 1.3	24.9 ± 1.9	52.8	15.4 ± 6.3	27.8
			20	18.5 ± 1.8	26.2 ± 3.8	41.6	33.7 ± 5.7	-58.3
			40	20.6 ± 1.9	25.0 ± 2.6	5.9	13.0 ± 4.3	38.9
24	4-chloro-benzo[d]-thiazole	-	10	18.6 ± 2.1	28.5 ± 1.0 *	53.2	5.8 ± 2.1 *	75.2
			20	17.5 ± 1.3	28.6 ± 0.6 *	63.4	21.5 ± 5.9	-0.9
			40	16.1 ± 1.7	22.4 ± 2.5	39.1	37.3 ± 9.1	-75.0
25	2-pyrimidine	-	10	17.3 ± 1.8	24.5 ± 3.7	41.6	11.2 ± 3.4	47.3
			20	15.3 ± 2.0	15.1 ± 1.9	-1.3	8.0 ± 2.1	43.9
			40	20.3 ± 1.6	24.7 ± 1.8	21.7	7.6 ± 5.2 *	64.2
26	4-OCH_3_-Ph	H	10	13.0 ± 1.9	16.6 ± 2.1	27.7	38.7 ± 6.6	-81.6
			20	11.4 ± 2.4	19.1 ± 1.6	67.5	43.7 ± 5.1	-105.3
			40	17.4 ± 1.3	23.8 ± 1.7	36.8	25.8 ± 7.7	-21.1
27	2-OCH_3_-Ph	H	10	18.9 ± 2.4	25.4 ± 2.1	34.4	29.2 ± 9.9	-37.1
			20	15.0 ± 2.3	20.6 ± 1.6	37.3	23.3 ± 6.4	-9.4
			40	13.8 ± 1.8	21.9 ± 1.4	58.7	11.7 ± 3.1	45.1
28	2-OCH_3_-Ph	C_2_H_5_	10	17.0 ± 1.5	24.1 ± 2.3	45.3	16.4 ± 3.3	22.9
			20	19.0 ± 1.5	27.3 ± 4.8	43.7	11.1 ± 2.4	47.8
			40	17.6 ± 1.4	26.4 ± 1.3	50.0	34.5 ± 10.2	-62.2
saline	-	-	-	16.4 ± 2.3	19.2 ± 1.8	17.1	21.3 ± 3.7	-
acetylsalicylic acid	-	-	100	-	-	-	5.2 ± 0.3 **	75.6
morphine	-	-	5	15.4 ± 1.6	36.3 ± 2.1 *	135.7	-	

^a^ Compound and acetylsalicylic acid were administered orally, morphine were administered subcutaneously; ^b^ Measured at 55.5 °C as licking latency (s) at various times after treatment; * *P* < 0.05, ** *P* < 0.01.

### 2.3. Acute Toxicity

Therefore, considering the results from compounds **10-28**, we concluded that compounds **10**, **18** and **19** showed more potent analgesic activity. We then assayed the acute toxicity of the compounds by determining their LD_50_ values (Table 2) [23]. Compounds **18** and **19** showed good safety profiles, even at the highest dose. No convulsions or tremors were detected in mice, during the 24 h observation. However, compound **10** showed side effects with the highest dose. 

**Table 2:** Acute toxicity LD_50_ of the compounds.

Compound ^a^	LD_50_ (mg/kg)
10	477.0 (357.2-637.1)
18	> 2,000
19	> 2,000

^a^ Compounds were administered orally.

### 2.4. Exploratory Locomotor Activity

Compounds that cause sedation will result in a reduction in spontaneous exploratory locomotor activity [24]. Pretreatment with 40, 80 and 160 mg/kg of compounds **18** and **19** did not reduce exploratory locomotor activity (Table 3). In comparison, clonazepam significantly reduced spontaneous locomotor activity. 

**Table 3:** Effects of compounds on exploratory locomotors activity.

Compound	Dose (mg/kg, po)	Average speed (cm/s)				
		Before treatment	After treatment			
			30 min	60 min	90 min	120 min
Control	-	8.48 ± 3.02	7.95 ± 2.85	7.64 ± 2.94	7.09 ± 2.89	7.98 ± 2.89
clonazepam	15	7.42 ± 2.62	3.28 ± 2.03 **	3.16 ± 2.32 **	3.41 ± 3.75 **	2.99 ± 1.89 **
18	40	8.56 ± 2.89	7.94 ± 2.50	7.75 ± 1.72	7.51 ± 1.74	7.88 ± 3.01
	80	7.63 ± 1.9	7.54 ± 3.05	7.03 ± 3.55	7.75 ± 2.95	7.46 ± 2.47
	160	7.49 ± 2.51	7.94 ± 2.72	7.56 ± 3.65	7.47 ± 2.17	6.98 ± 2.27
19	40	7.16 ± 2.07	6.75 ± 3.95	6.71 ± 2.02	5.62 ± 2.40	5.64 ± 3.13
	80	8.36 ± 3.23	7.83 ± 3.40	6.82 ± 3.26	7.44 ± 2.61	7.68 ± 2.43
	160	8.12 ± 2.61	6.36 ± 3.00	6.81 ± 2.84	6.88 ± 3.05	6.55 ± 2.99

^a^ Compound and clonazepam were administered orally; each value represents the mean of 10 mice; * P < 0.01, * P < 0.05 *vs*. before treatment.

Preliminarily, compounds were screened by both the writhing and hot plate tests at doses of 10, 20 and 40 mg/kg. Then, the compound with minimal toxicity base on the LD_50_ values was found. In addition, in order to examine the effect of synthesized compounds in the treatment of neuropathic pain, we refer to the dose of gabapentin (orally dose 40 mg/kg) [25,26,27].

### 2.5. Formalin Test

**Figure 2:**
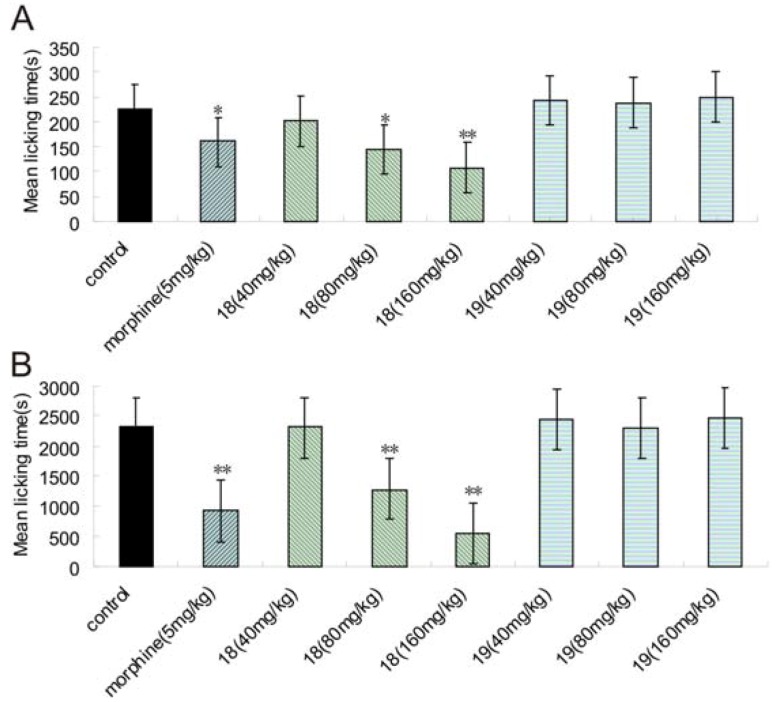
Effects of **18** and **19** administered po and morphine (ip) on the pain in rat. (A) the first phase (0-5 min); (B) the second phase (15-60 min). Each column represents mean ± S.E.M. of ten experimental values. * In comparison to the control group. * P < 0.05; ** P < 0.01.

The analgesic activity of compounds **18** and **19** were further investigated by the formalin test [28]. Compound **18** could significantly reduce pain responses compared to control groups in both phases at the dose of 80 and 160 mg/kg, whereas **19** was devoid of any efficacy in both phases (Figure 2 A, B).

### 2.6. Spared Nerve Injury (SNI) and Chronic Constriction Injury (CCI) Test

Currently available drugs for treating neuropathic pain are typically undesirable because of their intolerable side effects [8]. For example, gabapentin [1-(aminomethyl)cyclohexaneacetic acid] is considered to be the “gold standard” for a variety of neuropathic pains [28], but its efficacy is usually less than 50% [30], and it’s known to induce sedation at high doses [28]. To examine the potential therapeutic value of compounds **18** and **19** in the treatment of neuropathic pain, two well-known peripheral neuropathic pain models were investigated, including the spared nerve injury (SNI) [31] and chronic constriction injury (CCI) [32] models in rats.

In the SNI model, compound **18** (80, 160 mg/kg) significantly increased the mechanical allodynia when treated with single and repeated dose treatment (p < 0.05). After 13 days, compound **18** increased the pain threshold by 33.2% (80 mg/kg) and 40.4% (160 mg/kg) with single dose treatment, and by 29.6% (80 mg/kg) and 32.8% (160 mg/kg) with repeated dose treatment. In comparison, gabapentin increased the pain threshold by 33.7% and 42.7%, respectively (Table 4). These results suggested that the pain response inhibition of compound **18** was equivalent to that of gabapentin.

In the mechanical stimulation of CCI model, compound **18** (80 mg/kg) could significantly improve the mechanical allodynia in comparison to the model group with single and repeated dose treatment (p < 0.05). Compound **19** (80 mg/kg) only improved the mechanical allodynia with single dose treatment. After 13 days, compound 18 and gabapentin both significantly increased the pain threshold (Table 5).

**Table 4:** Effects of compounds on mechanical allodynia in the SNI model.

Compound	Dose (mg/kg, po)	Number of rats	Mechanical allodynia (g)			
			Baseline	Before administration (decreased rate of pain threshold %) ^a^	Single administration ^c ^(increased rate of pain threshold %) ^b^	Repeated administration ^c^ (increased rate of pain threshold %) ^b^
Sham	-	10	55.1 ± 4.4	49.7 ± 12.4 **	48.1 ± 11.1 **	55.8 ± 8.6 **
				9.7	-3.3	12.3
Control	-	10	61.86 ± 5.48	29.7 ± 4.3 ^##^	30.9 ± 4.8 ^##^	28.7 ± 3.8 ^##^
				52.0	3.9	-3.2
Gabapentin	40	10	60.9 ± 5.6	28.5 ± 5.3 ^##^	38.1 ± 5.4 *	40.6 ± 7.9 **
				53.3	33.7	42.7
18	40	10	60.3 ± 6.0	28.7 ± 4.1 ^##^	35.2 ± 8.6	30.5 ± 8.5
				52.3	22.3	5.9
	80	10	60.8 ± 3.2	28.4 ± 3.6 ^##^	37.8 ± 5.5 *	36.8 ± 4.8 *
				53.3	33.2	29.6
	160	10	63.4 ± 4.2	28.7 ± 5.1 ^##^	40.3 ± 5.4 **	38.1 ± 8.5 *
				54.7	40.4	32.8
19	40	10	61.8 ± 5.8	28.4 ± 7.2 ^##^	32.9 ± 9.1	34.5 ± 2.3
				54.1	15.8	21.6
	80	10	55.3 ± 13.3	29.0 ± 5.4 ^##^	33.9 ± 8.2	30.2 ± 6.7
				47.5	16.7	4.0
	160	10	58.8 ± 5.8	28.3 ± 5.6 ^##^	30.0 ± 5.4	33.1 ± 3.3
				51.8	5.8	16.8

^#^ p < 0.05; ^##^ p < 0.01 vs. baseline; * p < 0.05; ** p < 0.01 vs. control. ^a^ Decreased rate of pain threshold (%) = (pain threshold of 13th day-pain threshold of baseline) × 100% / pain threshold of baseline. ^b^ Increased rate of pain threshold (%) = (pain threshold of after administration-pain threshold of before administration) × 100% / pain threshold of before administration. ^c^ Each group was measured after first administration on the 14th day and the last administration on the 17th day, which was the result of single administration and repeated administration, respectively.

**Table 5:** Effects of compounds on mechanical allodynia in the CCI model.

Compound	Dose (mg/kg, po)	Number of rats	Mechanical allodynia (g)			
			Baseline	Before administration (decreased rate of pain threshold %) ^a^	Single administration ^c ^(increased rate of pain threshold %) ^b^	repeated administration ^c ^(increased rate of pain threshold %) ^b^
sham	-	10	56.0 ± 5.9	51.2 ± 9.0 **	53.4 ± 9.8 **	49.0 ± 7.3 **
				8.7	4.4	-4.2
control	-	10	58.3 ± 6.7	31.25 ± 5.1 ^##^	31.8 ± 5.5 ^##^	31.7 ± 6.4 ^##^
				46.5	2.1	1.73
Gabapentin	40	10	59.44 ± 6.20	31.4 ± 4.4 ^##^	39.1 ± 4.9 **	37.3 ± 7.5 *
				47.2	24.6	18.7
18	40	10	56.7 ± 4.7	34.1 ± 2.4 ^##^	36.2 ± 4.8	33. 6 ± 6.7
				39.9	6.3	-1.5
	80	10	58.7 ± 8.2	31.9 ± 3.5 ^##^	37.5 ± 8.3 *	38.0 ± 6.2 *
				45.68	17.6	19.2
	160	10	59.0 ± 4.4	32.1 ± 6.0 ^##^	35.9 ± 6.5	32.9 ± 4.0
				45.6	11.9	2.6
19	40	10	61.6 ± 4.9	31.3 ± 3.4 ^##^	32.9 ± 2.7	33.4 ± 3.4
				49.2	5.1	6.9
	80	10	56.4 ± 5.6	31.1 ± 4.8 ^##^	37.1 ± 3.5 *	32.0 ± 4.7
				44.9	19.3	2.8
	160	10	59.6 ± 5.2	31.1 ± 4.6 ^##^	32.1 ± 3.2	29.8 ± 7.2
				47.8	3.2	-4.3

^#^ p < 0.05; ^##^ p < 0.01 vs. baseline; * p < 0.05; ** p < 0.01 vs. control. ^a^ Decreased rate of pain threshold (%) = (pain threshold of 13th day-pain threshold of baseline) × 100% / pain threshold of baseline. ^b^ Increased rate of pain threshold (%) = (pain threshold of after administration-pain threshold of before administration) × 100% / pain threshold of before administration. ^c^ Each group was measured after first administration on the 14th day and the last administration on the 17th day, which was the result of single administration and repeated administration, respectively.

In the thermal stimulation of the CCI model, compound **18** increased the latency when treated with single and repeated doses (p < 0.05). After 13 days, compound **18** (40 mg/kg) increased latency by 53.5% and 66.4% with single and repeated dose treatment, significantly higher than the effectiveness of gabapentin (Table 6).

Overall, compound **18 **yielded high effectiveness in both the CCI and SNI model. Results also suggested that compound 18 was suitable for the treatment of neuropathic pain, which was equivalent to gabapentin without inducing severe sedative side effect.

**Table 6:** Effects of compounds on latency of thermal stimulation in the CCI model.

Compound	Dose (mg/kg, po)	Number of rats	Latency (s)			
			Baseline	Before administration (decreased rate of latency %) ^a^	Single administration ^c ^(increased rate of latency %) ^b^	Repeated administration ^c ^(increased rate of latency %) ^b^
Sham	-	10	14.7 ± 2.9	13.2 ± 2.7 *	13.41 ± 3.4 *	14.2 ± 4.1 *
				9.9	1.4	7.2
Control	-	10	15.1 ± 2.9	10.2 ± 3.1 ^##^	10.4 ± 3.4 ^##^	11.2 ± 3.3 ^##^
				32.5	2.5	9.5
Gabapentin	40	10	15.7 ± 3.6	11.9 ± 3.5 ^#^	17.9 ± 7.6 **	17.0 ± 13.9
				24.6	50.5	42.9
18	40	10	13.0 ± 1.7	8.7 ± 2.3 ^##^	13.4 ± 4.3 *	14.5 ± 3.9 *
				33.1	53.5	66.4
	80	10	15.5 ± 3.4	10.5 ± 3.7 ^##^	13.4 ± 4.1	11.2 ± 3.0
				32.34	27.7	6.7
	160	10	15.5 ± 5.5	9.3 ± 4.3 ^##^	18.6 ± 9.1 **	13.8 ± 7.3
				39.9	99.3	48.0
19	40	10	15.7 ± 2.4	9.6 ± 2.1 ^##^	11.5 ± 3.9	13.5 ± 8.1
				39.0	20.4	40.9
	80	10	12.9 ± 1.9	8.7 ± 2.7 ^##^	10.5 ± 2.9	10.5 ± 1.7
				33.1	21.2	21.4
	160	10	13.0 ± 1.9	9.2 ± 2.3 ^##^	11.5 ± 2.6	13.1 ± 4.2
				29.3	25.6	42.0

^#^ p < 0.05; ^##^ p < 0.01 vs. baseline; * p < 0.05; ** p < 0.01 vs. control. ^a^ Decreased rate of latency (%) = (latency of 13th day-latency of baseline) × 100% / latency of baseline. ^b^ Increased rate of latency (%) = (latency of after administration-latency of before administration) × 100% / latency of before administration. ^c^ Each group was measured after first administration on the 14th day and the last administration on the 17th day, which was the result of single administration and repeated administration, respectively.

### 2.7. Mechanism of Action

Due to its potent analgesic activity and minimal toxicity, compound **18** was selected for further investigation of its possible mechanism of action. The mechanism of pain transmission is complex because many different neuromodulators and receptors could be involved. Results indicated that compound **18** did not act on the opioid, 5HT_2A_ and 5HTU receptors. It was interesting to note that compound **18** had a high affinity for 5HT_1A_ (Table 7). The 5-HT_1A_ receptor functions as a somatodendritic autoreceptor that controls the release of serotonin in terminal areas. By preventing this inhibitory control of serotonin release, it is possible to enhance the analgesic effect of drugs that increase serotonin levels by facilitating both descending and ascending pathways involved in pain modulation [33]. As 5-HT_1A_ receptor agonist, 8-OH-DPAT could induce hyperalgesia in rats [34]. [(3-chloro-4-fluorophenyl)-[4-fluoro-4-{[(5-methylpyridin-2-ylmethyl)amino]methyl}piperidin-1-yl]methadone] (F13640) was a high-efficacy 5-HT_1A_ receptor agonist that exhibited an analgesic action in animal models of chronic, nociceptive and neuropathic pains [35,36,37]. In additional, a large number of arylpiperazines have potent affinity for 5-HT_1A_ receptors [38,39,40]. Thus, it appears that analgesia effect of compound 18 originates from its acting on 5HT_1A_ receptor.

### 2.8. Structure-Activity Relationships (SAR)

The following structure-activity relationships (SAR) could be drawn from the acetic acid induced writhing test and hot plate tests. The phenyl ring substituted with methoxy in the *ortho*-position (compound **10**) led to more activity than the *para*- and *meta*-positions (compounds **11,12**). This indicated that antinociceptive activity depended on the location of the substituent on the phenyl ring. Substitution with alkyl groups on the phenyl ring resulted in better analgesic activity, for example, 3-CF_3_-Ph (compound **18**) and 2,3-di-CH_3_-Ph (compound **19**) were observed to be the most active, with 78.7% and 75.2% inhibition. However, substitution with halogen groups was found to cause a dramatic decrease of activity, as demonstrated by compounds **13-17**. In the same subseries, replacement of the phenyl with aromatic heterocycle groups (compounds **20-25**) resulted in a complete loss of activity. A possible reason could be that the heterocycle groups impeded the phenyl ring from making a key hydrogen bond to a tightly bound water molecule. It seemed that phenyl ring was especially important for the analgesic activity. Finally, replacing carbonyl with a C=N-OR group (compounds **26-28**) resulted in the loss of activity, which indicated that the carbonyl was the key active site.

**Table 7:** Inhibition of the compounds for the relatively receptor.

Compound	Concentration (mol/L)	E_max10 μM_^a^ (%)					
		μ	δ	κ	5-HT_2A_	5-HT_1A_	5-HT uptake
Naloxone	10^-5^	100	100	100	-	-	-
Aripiprazole	10^-5^	-	-	-	100	-	-
5-HT	10^-5^	-	-	-	-	100	-
Duloxetine	10^-5^	-	-	-	-	-	90.6
w-conotoxinGVIA	10^-5^	-	-	-	-	-	-
18	10^-5^	18.1	0	2.9	57.7	96.9	59.3

^a^ Maximal inhibition effect at the highest tested concentration (E_max10 μM_).

## 3. Experimental

### 3.1. General

All the chemicals and solvents were purchased from commercial sources. Melting points were determined with a capillary melting point apparatus and are uncorrected. ^1^H-NMR spectra were recorded with an Avance 400 instrument (Bruker Biospin, version 002 with SGU). Chemical shifts are reported in ppm, using the solvent as internal standard. Coupling constants (*J* values) are given in Hertz (Hz). The mass spectra (MS) were recorded on an AMD-604 Mass Spectrometer operating at 70 eV.

#### 3.1.1. General Procedure for the Synthesis of Compounds **10-25** [20]

To a suspension of 2-chloro-1-phenylethanone (0.32 mmol), arylpiperazine (0.32 mmol) and anhydrous Na_2_CO_3_ (1.22 mmol) in anhydrous EtOH (5.0 mL) were added, and the resulting mixture was refluxed for 4 h. There after the mixture was filtered and the filtrate was evaporated to dryness under reduced pressure. The residues were recrystallized from isopropanol to give compounds **10-25**.

*2-(4-(2-Methoxyphenyl)piperazin-1-yl)-1-phenylethanone* (**10**): Yield 84.7%; Mp 102-104 °C; ^1^H- NMR (D_2_O) δ: 7.96 (d, 2H, *J* = 7.2 Hz, Ar-H), 7.72 (t, 1H, *J* = 7.2 Hz, Ar-H), 7.55 (t, 2H, *J* = 7.6 Hz, Ar-H), 7.16 (t, 1H, *J* = 7.6 Hz, Ar-H), 7.12 (d, 1H, *J* = 7.6 Hz, Ar-H), 7.05 (d, 1H, *J* = 7.6 Hz, Ar-H), 7.01 (t, 1H, *J* = 7.6 Hz, Ar-H), 5.00 (s, 2H, N-CH_2_-CO), 3.82 (s, 3H, -OCH_3_), 3.58 (br, 4H, piperazine-H), 3.39 (br, 4H, piperazine-H); MS (m/z) 311.3 ([M + H]^+^). 

*2-(4-(3-Methoxyphenyl)piperazin-1-yl)-1-phenylethanone* (**11**): Yield 84.7%; Mp 96-97 °C; ^1^H-NMR (CDCl_3_) δ: 8.01 (d, 2H, *J* = 7.6 Hz, Ar-H), 7.64 (t, 1H, *J* = 7.6 Hz, Ar-H), 7.53(t, 2H, *J* = 7.6 Hz, Ar-H), 7.10 (t, 1H, *J* = 8.0 Hz, Ar-H), 6.52 (dd, 1H, *J* = 7.6 and 2.0 Hz, Ar-H), 6.44 (t,1H, Ar-H), 6.36 (dd, 1H, Ar-H), 3.88 (s, 2H, N-CH_2_-CO), 3.71 (s, 3H, -OCH_3_), 3.13 (t, 4H, piperazine-H), 2.65 (t, 4H, piperazine-H); MS (ESI) m/z 311.3 ([M + H]^+^).

*2-(4-(4-Methoxyphenyl)piperazin-1-yl)-1-phenylethanone* (**12**): Yield 85%; Mp 107-109 °C; ^1^H-NMR (CDCl_3_) δ: 7.94 (d, 2H, *J* = 7.6 Hz, Ar-H ), 7.72 (t, 1H, *J* = 7.6 Hz, Ar-H), 7.55(t, 2H, *J* = 7.6 Hz, Ar-H), 7.12 (d, 2H, *J* = 8.8 Hz, Ar-H), 7.08 (d, 2H, *J* = 8.8 Hz, Ar-H), 5.00 (s, 2H, N-CH_2_-CO), 3.76 (s, 3H, -OCH_3_), 3.60 (br, 4H, piperazine-H), 3.50 (br, 4H, piperazine-H); MS (ESI) m/z 311.3 ([M + H]^+^).

*2-(4-(2-Chlorophenyl)piperazin-1-yl)-1-phenylethanone* (**13**): Yield 93%; Mp 89-90 °C; ^1^H-NMR (CDCl_3_) δ: 8.02 (d, 2H, *J* = 7.6 Hz, Ar-H), 7.64 (t, 1H, *J* = 7.6 Hz, Ar-H), 7.53 (t, 2H, *J* = 7.6 Hz, Ar-H), 7.39 (dd, 1H, *J* = 7.6 and 1.2 Hz, Ar-H), 7.28 (td, 1H, *J* = 7.6 and 1.2 Hz, Ar-H), 7.16 (dd, 1H, *J* = 7.6 and 1.2 Hz, Ar-H), 7.03 (td, 1H, *J* = 7.6 and 1.2 Hz, Ar-H), 3.93 (s, 2H, N-CH_2_-CO), 2.99 (br, 4H, piperazine-H), 2.71 (br, 4H, piperazine-H); MS (ESI) m/z 315.1 ([M + H]^+^).

*2-(4-(3-Chlorophenyl)piperazin-1-yl)-1-phenylethanone* (**14**): Yield 87%; Mp 115-116 °C; ^1^H-NMR (CDCl_3_) δ: 8.01 (d, 2H, *J* = 7.6 Hz, Ar-H), 7.64(t, 1H, *J* = 7.2 Hz, Ar-H), 7.53 (t, 2H, *J* = 8.0 Hz, Ar-H), 7.20 (t, 1H, *J* = 8.0 Hz, Ar-H), 6.93 (t, 1H, *J* = 2.0 Hz, Ar-H), 6.89 (dd, 1H, *J* = 7.6 and 1.2 Hz, Ar-H), 6.77(dd, 1H, *J* = 7.6 and 1.2 Hz, Ar-H), 3.92 (s, 2H, N-CH_2_-CO), 3.18 (t, 4H, *J* = 0.8 Hz, piperazine-H), 2.66 (t, 4H, *J* = 0.8 Hz, piperazine-H); MS (ESI) m/z 315.1 ([M + H]^+^).

*2-(4-(4-Chlorophenyl)piperazin-1-yl)-1-phenylethanone* (**15**): Yield 90%; Mp 137-138 °C; ^1^H-NMR (CDCl_3_) δ: 8.01 (d, 2H, *J* = 7.6 Hz, Ar-H), 7.64 (t, 1H, *J* = 7.2 Hz, Ar-H), 7.53(t, 2H, *J* = 7.6 Hz, Ar-H), 7.22 (t, 2H, *J* = 8.8 Hz, Ar-H), 6.94 (d, 2H, *J* = 8.8 Hz, Ar-H), 3.92 (s, 2H, N-CH_2_-CO), 3.14 (t, 4H, *J* = 0.8 Hz, piperazine-H), 2.66 (t, 4H, *J* = 0.8 Hz, piperazine-H); MS (ESI) m/z 315.1 ([M + H]^+^).

*2-(4-(2,3-Dichlorophenyl)piperazin-1-yl)-1-phenylethanone* (**16**): Yield 94%; Mp 106-107 °C; ^1^H-NMR (CDCl_3_) δ: 8.01 (d, 2H, *J* = 7.6 Hz, Ar-H), 7.59 (tt, 1H, *J* = 7.6 and 1.2 Hz, Ar-H), 7.47 (t, 2H, *J* = 7.6 Hz, Ar-H), 7.17-7.13 (m, 2H, Ar-H), 6.97 (dd, 1H, *J* = 5.6 and 2.8 Hz, Ar-H), 3.94 (s ,2H, N-CH_2_-CO) 3.16 (t, 4H, *J* = 4.8 Hz, piperazine-H), 2.85 (br, 4H, piperazine-H); MS (ESI) m/z 349.3 ([M + H]^+^).

*2-(4-(4-Fluorophenyl)piperazin-1-yl)-1-phenylethanone* (**17**): Yield 91%; Mp 127-128 °C; ^1^H-NMR (CDCl_3_) δ; 8.01 (d, 2H, *J* = 7.2 Hz, Ar-H ), 7.64 (t, 1H, *J* = 7.2 Hz, Ar-H), 7.53 (t, 2H, *J* = 7.6 Hz, Ar-H), 7.04 (t, 2H, *J* = 8.8 Hz, Ar-H), 6.95-6.93 (m, 2H, Ar-H), 3.92 (s, 2H, N-CH_2_-CO), 3.08 (t, 4H, *J* = 5.2 Hz, piperazine-H), 2.67 (t, 4H, *J* = 5.2 Hz, piperazine-H); MS (ESI) m/z 299.1([M + H]^+^).

*2-(4-(3-(Trifluoromethyl)phenyl)piperazin-1-yl)-1-phenylethanone* (**18**): Yield 91.3%; Mp 122-124 °C; ^1^H-NMR (MeOD) δ: 7.90 (d, 2H, *J* = 7.6 Hz, Ar-H), 7.58 (t, 1H, *J* = 7.6 Hz, Ar-H), 7.43(t, 2H, *J* = 7.6 Hz, Ar-H), 7.32 (t, 1H, *J* = 8.0 Hz, Ar-H), 7.14 (br, 2H, Ar-H), 7.04 (d, 1H, *J* = 8.0 Hz, Ar-H), 4.93 (s, 2H, N-CH_2_-CO), 3.79-3.14 (br, 8H, piperazine-H); MS (ESI) m/z 349.3 ([M + H]^+^).

*2-(4-(2,3-Dimethylphenyl)piperazin-1-yl)-1-phenylethanone* (**19**): Yield 92.5%; Mp 98-99 °C; ^1^H-NMR (MeOD) δ: 7.92 (d, 2H, *J* = 7.6 Hz, Ar-H), 7.59 (t, 1H, *J* = 7.6 Hz, Ar-H), 7.45(t, 2H, *J* = 7.6 Hz, Ar-H), 6.94 (t, 1H, *J* = 7.6 Hz, Ar-H), 6.86 (d, 1H, *J* = 7.6 Hz, Ar-H), 6.82 (d, 1H, *J* = 7.6 Hz, Ar-H), 4.94 (s, 2H, N-CH_2_-CO), 3.59-3.09 (br, 8H, piperazine-H), 2.12 (s, 6H, Ar-CH_3_); MS (ESI) m/z 309.3 ([M + H]^+^).

*2-(4-(6-Methoxybenzo[d]thiazol-2-yl)piperazin-1-yl)-1-phenylethanone* (**20**): Yield 85%; Mp 159-161 °C; ^1^H-NMR (CDCl_3_) δ: 7.92 (d, 2H, *J* = 7.6 Hz, Ar-H), 7.58 (tt, 1H, *J* = 7.2 and 1.2 Hz, Ar-H), 7.47 (t, 2H, *J* = 7.6 Hz, Ar-H), 7.45 (s, 1H, Ar-H), 7.15 (d, 1H, *J* = 2.4 Hz, Ar-H), 6.90 (dd, 1H, *J* = 7.6 and 2.4 Hz, Ar-H), 3.90 (s, 2H, N-CH_2_-CO), 3.82 (s, 3H, -OCH_3_), 3.68 (t, 4H, *J* = 5.2 Hz, piperazine-H), 2.76 (t, 4H, *J* = 5.2 Hz, piperazine-H); MS (ESI) m/z 368.3 ([M + H]^+^).

*2-(4-(6-methylbenzo[d]thiazol-2-yl)piperazin-1-yl)-1-phenylethanone* (**21**): Yield 80%; Mp 155.5-157.0 °C; ^1^H-NMR (CDCl_3_) δ: 8.00 (d, 2H, *J* = 7.6 Hz, Ar-H), 7.64(t, 1H, *J* = 7.6 Hz, Ar-H), 7.56-7.51 (m, 3H, Ar-H), 7.34 (d, 1H, *J* = 8.4 Hz, Ar-H), 7.08 (dd, 1H, *J* = 7.6 and 0.8 Hz, Ar-H), 3.97 (s, 2H, N-CH_2_-CO), 3.55 (t, 4H, *J*=1.2 Hz, piperazine-H), 2.68 (t, 4H, *J* = 1.2Hz, piperazine-H), 2.33 (s, 3H, Ar-CH_3_); MS (ESI) m/z 352.3 ([M + H]^+^).

*2-(4-(4-Methylbenzo[d]thiazol-2-yl)piperazin-1-yl)-1-phenylethanone* (**22**): Yield 82%; Mp 139-141 °C; ^1^H-NMR (CDCl_3_) δ: 8.00 (m, 2H, Ar-H), 7.58(tt, 1H, *J* = 7.2 and 1.2 Hz, Ar-H), 7.49-7.43 (m, 3H, Ar-H), 7.12 (d, 1H, *J* = 7.2 Hz, Ar-H), 6.98 (t, 1H, *J* = 7.6 Hz, Ar-H), 3.91 (s, 2H, N-CH_2_-CO), 3.74 (t, 4H, *J* = 5.2 Hz, piperazine-H), 2.77 (t, 4H, *J* = 5.2 Hz, piperazine-H), 2.56 (s, 3H); MS (ESI) m/z 352.3 ([M + H]^+^).

*2-(4-(6-Chlorobenzo[d]thiazol-2-yl)piperazin-1-yl)-1-phenylethanone* (**23**): Yield 81%; Mp 172-173.5 °C; ^1^H-NMR (CDCl_3_) δ: 7.98 (d, 2H, *J* = 7.6 Hz, Ar-H), 7.60 (tt, 1H, *J* = 7.2 and 1.2 Hz, Ar-H), 7.57 (d, 1H, *J* = 2.0 Hz, Ar-H), 7.48 (t, 2H, *J* = 7.6Hz, Ar-H), 7.45 (d, 1H, *J* = 8.8 Hz, Ar-H), 7.25 (dd, 1H, *J* = 8.8 and 2.0 Hz, Ar-H), 3.97 (s, 2H, N-CH_2_-CO), 3.76 (t, 4H, *J* = 5.2 Hz, piperazine-H), 2.85 (br, 4H, piperazine-H); MS (ESI) m/z 372.3 ([M + H]^+^).

*2-(4-(4-Chlorobenzo[d]thiazol-2-yl)piperazin-1-yl)-1-phenylethanone* (**24**): Yield 81%; Mp 146-148 °C; ^1^H-NMR (400 MHz, CDCl_3_) δ: 7.98 (d, 2H,*J* = 7.6 Hz, Ar-H), 7.59 (t, 1H, *J* = 7.6 Hz, Ar-H), 7.49-7.42 (br, 3H, Ar-H), 7.31 (dd, 1H, *J* = 7.6 and 1.2Hz, Ar-H), 6.98 (t, 1H, *J* = 8.0 Hz, Ar-H), 3.77 (s ,2H, N-CH_2_-CO), 3.72(t, 4H, *J* = 5.2 Hz, piperazine-H), 2.77 (t, 4H, *J* = 5.2 Hz, piperazine-H); MS (ESI) m/z 372.3 ([M + H]^+^).

*2-(4-(Pyrimidin-2-yl)piperazin-1-yl)-1-phenyl ethanone* (**25**): Yield 90%; Mp 156-158 °C; ^1^H-NMR (D_2_O) δ: 8.51 (d, 2H, *J* = 5.2 Hz, Ar-H), 7.91 (d, 2H, *J* = 7.6 Hz, Ar-H), 7.68 (t, 1H, *J* = 7.6 Hz, Ar-H), 7.51 (t, 2H, *J* = 8.0 Hz, Ar-H), 6.97 (t, 1H, *J* = 5.2 Hz, Ar-H), 5.00 (s, 2H, N-CH_2_-CO), 4.14-2.89 (br, 8H, piperazine-H); MS (ESI) m/z 283.2([M + H]^+^).

#### 3.1.2. General Procedure for the Synthesis of Compounds **26-28**

A mixture of hydroxylamine hydrochloride or N-ethylhydroxylamine hydrochloride (0.012 mol), compound **10** or **12** and 20% aqueous sodium hydroxide (12 mL) in water (10 mL) was heated at 100 °C for 1 h. Reaction was cooled to room temperature, and then the precipitate is collected by filtration and washed with 50 mL water, gave the corresponding oxime **26-28**.

*2-(4-(4-Methoxyphenyl)piperazin-1-yl)-1-phenylethanone oxime* (**26**): Yield 65%; Mp 168-170 °C; ^1^H-NMR (CDCl_3_) δ: 11.45 (s, 1H, -NOH), 7.77 (dd, 2H, *J* = 7.6 and 1.2 Hz, Ar-H), 7.39-7.34 (br, 3H, Ar-H), 6.84 (d, 2H, *J* = 9.2 Hz, Ar-H), 6.76 (d, 2H, *J* = 9.2 Hz, Ar-H), 3.68 (s, 2H, N-CH_2_-CO), 3.66 (s, 3H, -OCH_3_), 2.93 (br, 4H, piperazine-H), 2.57 (br, 4H, piperazine-H); MS (ESI) m/z 326.2 ([M + H]^+^). 

*2-(4-(2-Methoxyphenyl)piperazin-1-yl)-1-phenylethanone oxime* (**27**): Yield 65%; Mp 129-131 °C; ^1^H-NMR (CDCl_3_) δ: 12.70 (s, 1H, -NOH), 7.86-7.84 (br, 2H, Ar-H), 7.48(t, 3H, *J* = 3.2 Hz, Ar-H), 6.97-6.89 (m, 4H, Ar-H), 4.53 (s, 2H, N-CH_2_-CO), 3.77 (s, 3H, OCH_3_), 3.45-3.05 (br, 8H, piperazine-H); MS (ESI) m/z 326.2 ([M + H]^+^).

*2-(4-(2-Methoxyphenyl)piperazin-1-yl)-1-phenylethanone ethyl oxime (28)*: Yield 70%; Mp 145-147 °C; ^1^H-NMR (CDCl_3_) δ: 7.84 (dd, 2H, *J* = 6.8 and 2.0 Hz, Ar-H), 7.50-7.46 (br, 3H, Ar-H), 7.04-6.87 (m, 4H, Ar-H), 4.54 (s, 2H, N-CH_2_-CO), 4.34(q, 2H, *J* = 7.2Hz, N-OCH_2_CH_3_), 3.78 (s, 3H, OCH_3_), 3.45-3.09 (br, 8H, piperazine-H), 1.34 (t, 3H, N-OCH_2_CH_3_); MS (ESI) m/z 354.2 ([M + H]^+^). 

### 3.2. Pharmacology

#### 3.2.1. Animals

Chinese Kun Ming (KM) Mice (20 ± 2.0 g) and Sprague-Dawley (SD) rats (250 ± 5.0 g) were used as experimental animals in this study. Animals were housed under standardized conditions for light and temperature and received standard rat chow and tap water *ad libitum*. Animals were randomly assigned to different experimental groups, each kept in a separate cage.

##### 3.2.1.1. Acetic Acid-Induced Abdominal Constrictions Assay [21]

Mice were divided into groups of ten each, to test the abdominal constriction response caused by intraperitoneal injection of diluted acetic acid (0.6%, 0.4 mL). Compounds were administered orally (10, 20, 40 mg/kg) as a suspension in 5% saline (vehicle). Acetylsalicylic acid (100 mg/kg, po) was used as standard drug under same conditions. Control animals received an equal volume of vehicle. Test group mice received acetic acid 1 h after drug treatment. The number of constrictions per animal was recorded for 15 minutes. For scoring purposes, a writhe was indicated by stretching of the abdomen with simultaneous stretching of at least one hind limb. Analgesic activity was expressed as percentage of inhibition of constrictions in comparison to control group:


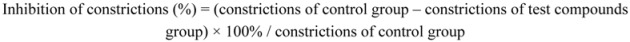


##### 3.2.1.2. Hot Plate Test [22]

The mice were treated with saline solution, morphine (5 mg/kg, sc) or test compounds (10, 20, 40 mg/kg, po), and placed individually on a hot-plate maintained at 55 ± 1 °C. The time between placing the animal on the hot-plate and the occurrence of either the licking of the hind paws, shaking the paw or jumping off the surface was recorded as response latency. Mice with baseline latencies of < 5 or > 30 s were eliminated from the study, and the cut-off time for the hot-plate latencies was set at 60 s. The animals were treated 60min before the assay:


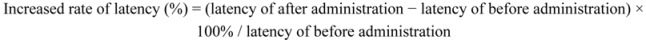


##### 3.2.1.3. Acute Toxicity Study (LD_50_) [23]

The test compounds were investigated for their acute toxicity (LD_50_) in mice. The test compounds were given orally at different dose levels in separate groups of animals. After 24 h of drug administration, percent mortality in each group was observed. The LD_50 _calculations were done by the Bliss method.

##### 3.2.1.4. Exploratory Locomotor Activity [24]

Exploratory locomotor activity was assessed in 14 automated activity frames equipped with infrared photobeam emitters and sensors. To assess drug effect on exploratory locomotor activity, the mice were transferred to new home cages immediately before test start and activity was measured for 30 min. Test or reference compounds were orally administered 30 min before test start at the following doses: 18 and 19, 40, 80, 160 mg/kg; clonazepam: 15.0 mg/kg. The average speed was measured before and 30, 60, 90 and 120 min after treatment.

##### 3.2.1.5. Formalin Test [28]

Male rats, 10/group, were treated with the tested compounds (40, 80, 160 mg/kg, po) and morphine (5 mg/kg, ip), and 1 h later, a 5% formalin solution (50 μL) was injected subcutaneously into the dorsal surface of their right hind paws. The formalin induced a typical licking or biting of the injected paw (flinching behavior). The animals were placed in a transparent chamber, The total time (s) spent licking the injected paw during periods of 0-5 min and 15-60 min after formalin injection was measured as an indicator of nociceptive behavior.

### 3.3. Spared Nerve Injury (SNI) Neuropathy Assay

#### 3.3.1. Group and Design

Rats were randomly divided into several groups (10/group): sham, control, gabapentin (40 mg/kg) and compounds group (40, 80 and 160 mg/kg). Pain threshold base value of each group were measured 1-2 days before operation with the values of two days were picked. The pain threshold values were measured again 13 days after the operation to check if the model were successful. Gabapentin, 18 and **19** were dosed orally twice a day for four days (14th, 15th, 16th and 17th day). The behavior test was performed 1 h after administration. Each group was measured after the first administration on the 14th day and the last administration on the 17th day, which was the result of single administration and repeated administration, respectively.

#### 3.3.2. Surgery [31,41]

Rats were anaesthetized with 10% chloral hydrate and the skin of the lateral left thigh was incised. The cranial and caudal parts of the biceps femoris muscle were separated and held apart by a retractor to expose the sciatic nerve and its three terminal branches: the sural, common peroneal and tibial nerves. The common peroneal and the tibial nerves were tight-ligated with 5.0 silk and sectioned distal to the ligation, removing 2-4 mm of the distal nerve stump. Any stretching or contact with the intact sural nerve was avoided. The muscle and skin were closed in two layers and the skin sutured together with hidden stitches to avoid any opening of the wound by biting. Sham controls involved exposure of the sciatic nerve and its branches without any lesion.

#### 3.3.3. Mechanical Withdrawal Threshold [41]

Rats were placed in a transparent plexiglass box (22 cm × 12 cm × 22 cm), with a metal mesh floor to allow for stimulation of the lateral plantar surface of the paw (innervated by the spared sural nerve). The animals were adapted to the testing situation for at least 30 min before the session started. A set of von Frey monofilaments was used to test the mechanical withdrawal threshold of the hindpaws. The monofilaments were applied in increasing force until the rat withdrew the paw. For each measurement, the paw was sampled four times and a mean calculated.

### 3.4. Chronic Constriction Injury (CCI) Neuropathy Assay

#### 3.4.1. Group and Design

Rats were randomly divided into several groups (10/group): sham, control, gabapentin (40 mg/kg) and compounds group (40, 80 and 160 mg/kg). Pain threshold base value of each group were measured 1-2 days before operation with the values of two days were picked. The Pain threshold values were measured again 13 days after the operation to check if the model were successful. Gabapentin, 18 and 19 were dosed orally twice a day for four days (14th, 15th, 16th and 17th day). The behavior test after 1 h of administration. Each group was measured after first administration on the 14th day and the last administration on the 17th day, which was the result of single administration and repeated administration, respectively.

#### 3.4.2. Surgey [32]

Rats were anaesthetized with chloral hydrate. The common sciatic nerve is exposed at the level of the mid thigh by blunt dissection through the biceps femoris. A section of nerve proximal to the sciatic trifucation, about the nerve was freed of adhering tissue and four ligatures (4/0 silk tread) were tied loosely around it with about 1mm spacing. The length of the nerve thus affected was 1 cm long. The incision is closed in layers and the animals are allowed to recover. Surgery for the sham condition involved exposing the sciatic nerve, but the nerve was not isolated from surrounding tissue.

#### 3.4.3. Behavioral Testing

##### 3.4.3.1. Mechanical Withdrawal Threshold (MWT) [42]

The rats were placed in a transparent plexiglass box (22 cm × 12 cm × 22 cm), with a 5 × 5 mm wire-mesh grid floor to allow for stimulation of the lateral plantar surface of the paw(innervated by the spared sural nerve). The animals were adapted to the testing situation for at least 30 min before the session started. A set of von Frey monofilaments was used to test the mechanical withdrawal threshold of the hindpaws.The monofilaments were applied in increasing force until the rat withdrew the paw. For each measurement, the paw was sampled four times and a mean calculated. At least 3 min elapsed between.

##### 3.4.3.2. Thermal Withdrawal Latency (TWL) [43]

The rats were placed in a clear plastic chamber (22 cm × 12 cm × 22 cm) with a 3 mm glass floor and allowed to acclimate to their environment for 30 min before testing. A radiant heat source (BME-410A) is aimed at the mid-plantar hindpaw (sciatic nerve territory) through the glass floor. The latencies for the withdrawal reflex in both paws are recorded. A 60 s cut off is used to prevent permanent injury to the skin. For each measurement, the paw was sampled four times and a mean calculated. At least 3 min elapsed between tests.

### 3.5. 5-HT_1A_ Binding Assay [44]

Cerebral cortex was homogenized in 20 volumes of ice-cold Tris-HCl buffer (50 mM, pH 7.7 at 22 °C) using ULTRA TURAX homogeniser, and was then centrifuged at 32,000 g for 10 min. The supernatant fraction was discarded, and pellet was resuspended in the same volume of Tris-HCl buffer and was then centrifuged. Before the third centrifugation, the samples were incubated at 37 °C for 10 min. The final pellet was resuspended in Tris-HCl buffer containing 10 μM pargyline, 4 mM CaCl_2_ and 0.1% ascorbic acid. One milliliter of the tissue suspension (9 mg of wet weight), 100 μL of 10 μM serotonin (for unspecific binding), 100 μL of [^3^H]-8-OH-DPAT and 100 μL of analyzed compound were incubated at 37 °C for 15 min. The incubation was followed by a rapid vacuum filtration through Whatman GF/B glass filters, and the filters were washed twice with 5 mL cold buffer and transferred to scintillation vials. Scintillation fluid (3.0 mL) was added and the vials were counted the next day using a Beckman LS 6500 liquid scintillation counter. Each experiment was performed in duplicate:


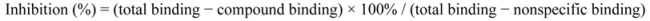


### 3.6. 5-HT_2A_ Binding Assay [45]

Cerebral cortex was homogenized in 20 volumes of ice-cold Tris-HCl buffer (50 mM, pH 7.7 at 22 °C) using ULTRA TURAX homogeniser, and centrifuged at 32,000 g for 20 min. The resulting pellet was resuspended in the same quantity of the buffer, preincubated at 37 °C for 10 min and centrifuged for 20 min. The final pellet was resuspended in 50 volumes of the same buffer. One milliliter of the tissue suspension, 100 μL of 1 μM methisergide, 100 μL of [^3^H]-ketanserin and 100 μL of the analyzed compound were incubated at 37 °C for 20 min, followed by a rapid vacuum filtration through Whatman GF/B glass filters, The filters were washed twice with 5 mL cold buffer and transferred to scintillation vials. Liquid scintillation (3.0 mL) was added and the vials were counted the next day using a Beckman LS6500 liquid scintillation counter. The final [^3^H]-ketanserin concentration was 1 nM, and the concentrations of the analyzed compounds 10 μM. Each experiment was performed in duplicate:


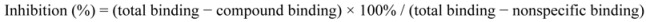


### 3.7. 5-HT Uptake Binding Assay [46]

A human carcinoma cell line possessing low endogenous levels of the 5-HT transporter were seeded into 96-well plates and treated with staurosporine at least 18 h prior to assay. On the day of assay, vehicle, excess of fluoxetine, or test compound was added to various wells on the plate. All wells then received [^3^H]-5-HT and were incubated at 37 °C for 5 min. The wells are then washed with ice cold 50 mM Tris-HCl (pH 7.4) buffer and aspirated to remove free [^3^H]-5-HT. An amount of 25 µL of 0.25 M NaOH is then added to each well to lyse the cells and 75 µL scintillation fluid added and bound radioactivity was quantitated using a Beckman LS6500 liquid scintillation counter. Tubes with vehicle represent total possible uptake, and radioactivity counted intubes with duloxetine represent nonspecific binding/uptake and is subtracted from the total possible uptake to give total possible specific uptake. This nonspecific binding (usual low in number) is then subtracted from the counts obtained in wells with various test compounds to give specific uptake in the presence of drug. Specific uptake is then expressed as a % of control values.

### 3.8. μ-Opioid Receptor Binding Assay [47]

A suspension of membranes from human μ-opioid receptor-expressing CHO cells in 50 mM Tris-HCl buffer (pH 7.4) containing 5 mM MgCl_2_ and 10% sucrose was incubated at room temperature for 2.5 h with 0.33 nM [^3^H]-diprenorphine and 10 μΜ of compounds .The membranes were collected by filtration using Whatman GF/B glass filters, and radio-activity was counted with a Beckman LS 6500 liquid scintillation counter. Nonspecific binding (6.4%) was determined with 10 μM naloxone. Specific binding was calculated by subtracting nonspecific binding from the total binding. Each experiment was performed in duplicate.

### 3.9. k-Opioid Receptor Binding Assay [47]

A suspension of membranes from human *k*-opioidreceptor-expressing HEK 293 cells in 50 mM Tris buffer (pH 7.4) containing 5 mM MgCl_2_, 1 mM EDTA, and 10% sucrose was incubated at room temperature for 1 h with 0.41 nM [^3^H]-diprenorphine and 10 μΜ of compounds.The membranes were collected by filtration using Whatman GF/B glass filters, and radioactivity was counted with a Beckman LS6500 liquid scintillation counter. Nonspecific binding (2.3%) was determined with 100 μM naloxone. Specific binding was calculated by subtracting nonspecific binding from the total binding.

### 3.10. δ-Opioid Receptor Binding Assay [47]

A suspension of membranes from human *k*-opioidreceptor-expressing receptor-expressing CHO cells in 50 mM Tris-HCl buffer (pH 7.4) containing 5 mM MgCl_2 _and 10% sucrose was incubated at room temperature for 1 h with 0.55 nM [^3^H]-diprenorphine and 10 μΜ of compounds. The membranes were collected by filtration using Whatman GF/B glass filters, and radioactivity was counted with a Beckman LS6500 liquid scintillation counter. Nonspecific binding (2.3%) was determined with 100 μM naloxone. Specific binding was calculated by subtracting nonspecific binding from the total binding.

### 3.11. Statistical Analysis

The results are presented as means ± SEM. The statistical significance between the groups was determined by analysis of variance followed by Dunnett’s multiple comparison test. P-values of less than 0.05 were considered indicative of significance.

## 4. Conclusions

In conclusion, the proposed piperazine derivatives, compounds **10-28,** possessed a broad spectrum of analgesic activity. Compounds **18** and **19** showed remarkable analgesic activity in both the writhing and hot plate tests. They have also also shown good safety margins without sedative side effects. Furthermore, compound **18** could significantly reduce licking time in the formalin-induced model, and was even more active than morphine. As a non-opioid analgesic agent with high efficacy, the effectiveness of compound **18** is equal to that of gabapentin in the neuropathic pain models. Thus, our results suggested that compound **18** may be a good drug candidate for effective neuropathic pain treatment.
